# Adherence to prenatal iron-folic acid supplementation in low- and middle-income countries (LMIC): a protocol for systematic review and meta-analysis

**DOI:** 10.1186/s13643-018-0774-x

**Published:** 2018-07-25

**Authors:** Mohammed Akibu, Tesfalidet Tekelab, Abdella Amano, Merga Besho, Stephanie Grutzmacher, Mesfin Tadese, Tesfa Dejenie Habtewold

**Affiliations:** 1 Institute of Medicine and Health Science, Debre Berhan University, Debre Berhan, Ethiopia; 2 Research Centre for Generational Health and Ageing at the Hunter Medical Research Institute, University of Newcastle, Callaghan, Australia; 3 College of Medicine and Health Science, Wollaga University, Nekemte, Ethiopia; 4 College of Medicine and Health Science, School of Public Health, Hawassa University, Hawassa, Ethiopia; 5 School of Biological and Population Health Sciences, Oregon State University, Corvallis, USA; 6 Department of Epidemiology, University Medical Center Groningen, University of Groningen, Groningen, The Netherlands; 7 Department of Midwifery, Institute of Medicine and Health Science, Debre Berhan University, P.O. Box 445, Debre Berhan, Ethiopia

**Keywords:** Folic acid, Folate, Iron, Medication adherence, Patient compliance, Prenatal Nutritional Physiological Phenomena, Prenatal care, Pregnancy

## Abstract

**Background:**

Daily iron-folic acid supplementation reduces anemia and various adverse obstetric outcomes such as preterm delivery, low birthweight, hemorrhage, and perinatal and maternal morbidity and mortality. However, its supplementation has not been successful that attributed to several determinants including poor adherence. Therefore, we aimed to conduct a systematic review and meta-analysis on the prevalence and determinants of adherence to prenatal iron-folic acid supplementation in low- and middle-income countries. In addition, we will develop a conceptual framework in the context of low- and middle-income countries (LMIC).

**Methods/design:**

We will search PubMed, MEDLINE, EMBASE, EBSCO, Web of Science, SCOPUS, WHO Global Index Medicus, and African Journals Online (AJOL) databases to retrieve relevant literatures. Observational (i.e., case-control, cohort, cross-sectional, survey, and surveillance reports) and quasi-randomized and randomized controlled trial studies conducted in LMIC will be included. The Newcastle-Ottawa Scale (NOS) and Joanna Briggs Institute (JBI) critical appraisal checklist will be used to assess the quality of observational and randomized controlled trial studies respectively. The pooled prevalence and odds ratio of determinants of adherence will be generated using a weighted inverse-variance meta-analysis model. Statistical heterogeneity among studies will be assessed by Cochran’s *Q χ*^2^ statistics and Higgins (*I*^2^ statistics) method. The result will be presented using forest plots and Harvest plots when necessary. Furthermore, we will perform Jackknife sensitivity and subgroup analysis. Data will be analyzed using comprehensive meta-analysis software (version 2).

**Discussion:**

Contemporary evidence about the prevalence and determinants of adherence in LMIC will be synthesized to generate up-to-date knowledge. To our knowledge, this is the first systematic review. It would have substantial implications for researchers, clinicians, and policymakers for optimizing maternal and child health outcomes in LMIC.

**Systematic review registration:**

The protocol has been registered on International Prospective Register of Systematic Review (PROSPERO), University of York Center for Reviews and Dissemination (https://www.crd.york.ac.uk/), registration number CRD42017080245.

**Electronic supplementary material:**

The online version of this article (10.1186/s13643-018-0774-x) contains supplementary material, which is available to authorized users.

## Background

Anemia is a global public health problem affecting nearly two billion people [1]. Despite vulnerability across the population, anemia is prevalent in pregnant woman (> 40%) and young children because of increased demand and iron-folic acid deficiency [2]. Iron-folic acid deficiency increases the risk of blood loss during labor, maternal mortality, preterm delivery, low birthweight, and perinatal mortality [3, 4]. Thus, to prevent these poor health outcomes, the World Health Organization (WHO) has recommended that all pregnant women take a standard dose of 60 mg of elemental iron along with 400 μg of folic acid daily for the first 6 months. Additionally, in areas where the prevalence of anemia is over 40%, the WHO recommends postpartum supplementation for 3 months [2].

A systematic review and meta-analysis of maternal anemia in lower and middle-income countries (LMIC) showed that 12, 19, and 18% of low birthweight, preterm births, and perinatal mortality are associated with maternal anemia respectively [5]. Accumulated body of evidence shows that prenatal iron-folic acid supplementation reduces maternal anemia and associated adverse perinatal outcomes, such as low birthweight, and maternal and newborn mortality [6, 7].

In LMIC, high proportion of pregnant women suffered from anemia mainly due to poor adherence to the daily iron-folic acid regimen [1, 8]. Furthermore, various individual studies shows that poor adherence is common in LMIC [9–11]. In our review, adherence is defined as taking at least five iron-folic acid tablets per week [2] or percentage of women who consumed more than 70% of the recommended daily dose [12].

Generally, pregnant women living in LMIC need a support for initiating and maintaining optimal adherence to the recommended iron-folic acid supplementation [13]. Therefore, this systematic review and meta-analysis aimed to (1) investigate the level of adherence to prenatal iron-folic acid supplementation, (2) identify its determinants, and (3) develop PRECEDE-PROCEED adherence conceptual framework in the context of LMIC.

## Methods/design

### Reporting of the review findings

This protocol has been written in accordance with the recommendation of Preferred Reporting Items for Systematic Review and Meta-Analysis (PRISMA-P) 2015 statement [14]. The PRISMA-P Elaboration and Explanation document is also used to develop the protocol [15] (Additional file 1). The protocol has been registered on International Prospective Register of Systematic Review (PROSPERO), University of York Center for Reviews and Dissemination [https://www.crd.york.ac.uk/], registration number CRD42017080245. Preferred Reporting Items for Systematic review (PRISMA-2009) statement will be used to report the findings. Furthermore, PRISMA flow diagram will be used to illustrate study screening and selection process.

### PECO search guide

- P-population: pregnant mothers who have received prenatal iron-folic acid
- E-exposure: determinants of adherence (e.g., income level, educational status) and interventions and intervention characteristics/components (e.g., how the supplements are delivered to the women, whether they receive instruction on how and why they should take them, reminders that are sent via text message) that are associated with prenatal iron-folic adherence. PRECEDE-PROCEED conceptual framework [10] will be used to select potential exposures/determinants
- C-comparison: the reported reference group for each determinant in each study (e.g., adherence in pregnant women with high educational status versus adherence in women with low educational status)
- O-outcome: adherence to iron-folic acid supplementation

### Data source and search strategy

PubMed, MEDLINE, EMBASE, EBSCO, Web of Science, and SCOPUS databases will be searched to retrieve all available studies. We will also extend our searching to WHO Global Index Medicus and African Journals Online (AJOL). Cross-references of included studies will be hand-searched as well to access additional relevant articles that may have been missed in the search. In addition, we will search existing reviews and perform citing studies/snowballing search in PubMed and SCOPUS databases to screen all studies that cited included studies. Likewise, a search for gray literature will be conducted using Google Scholar and through browsing Hinari (http://www.who.int/hinari/en/), Carolina digital repository (https://cdr.lib.unc.edu/), and SpringerOpen-Open repository (https://www.springeropen.com/get-published/indexing-archiving-and-access-to-data/open-repository). Medical subject headings (Mesh), keywords, and free-text words were identified for selected PECO components. “OR”, “AND”, and “NOT” Boolean operators were used to combine search terms. Moreover, we will contact Cochrane Pregnancy and Childbirth Group, WHO headquarters and regional offices, the nutrition section of the United Nations Children’s Fund (UNICEF), the World Food Program (WFP), and US Agency for International Development (USAID) micronutrient program to identify additional studies [16]. The search strategy for PubMed database has been designed in consultation with medical information specialist and supplemented with this protocol (Additional file 2). The Peer Review of Electronic Search Strategies (PRESS) 2015 guideline statement is followed to prepare the search strategy [17].

### Eligibility criteria

The inclusion criteria are (1) observational (i.e., case-control, cohort, cross-sectional, survey, and surveillance reports) and quasi-randomized and randomized controlled trial studies, (2) studies conducted in LMIC, and (3) studies that reported the prevalence and/or least adjusted determinants of adherence. The most up-to-date World Bank country classification, when our review is published, will be used to categorize LMIC [18]. The search will not be restricted to any language and publication year. Qualitative studies that thematically analyzed the determinants of adherence will be included. Studies conducted in study populations other than pregnant women will be excluded. Moreover, case reports and expert opinion will be excluded. We will pilot the eligibility criteria in at least 200 references and double-check if they allow unambiguously included or excluded studies.

### Selection of studies

Covidence web-based software will be used to remove duplicated articles and carry out all of the screening processes. First, articles will be assessed for inclusion through a title and abstract review by two independent reviewers. Disagreement will be solved by consensus; a third reviewer will be invited in case of persistent contradiction. Second, potentially eligible studies will undergo full-text review to determine if they satisfy the criteria set for inclusion. We will do a full-text review in duplicates and clearly document reasons for inclusion and exclusion. Finally, data will be extracted from all articles that meet the inclusion criteria.

### Data extraction

Data will be extracted using the Joanna Briggs Institute (JBI) data extraction form for experimental/observational studies (Additional file 3) [19]. The data extraction form will be pre-tested with 3–5 eligible studies. Two reviewers will independently extract all relevant information including study setting, sample size, prevalence of adherence to iron-folic acid supplementation, least-adjusted determinants, and source of funding. The prevalence of adherence to iron-folic acid will be extracted only if reported and/or estimated based on experts’ opinion or previously published studies or guidelines. In case of incomplete data, the corresponding author(s) will be contacted to find full information. Disagreement between reviewers will be resolved by consensus.

### Quality assessment

The quality of all included studies will be rigorously assessed by two independent reviewers. The Newcastle-Ottawa Scale (NOS) will be used to assess the quality of cohort and case-control articles [20]. Similarly, cross-sectional studies will be examined using NOS adapted for cross-sectional studies. NOS has a good inter-rater reliability and validity [21]. The NOS criteria and its rating system have been published elsewhere [22]. The Joanna Briggs Institute (JBI) critical appraisal checklist will be used to assess the quality of quasi-randomized controlled trials [23].

### Data synthesis and analysis

Data will be analyzed using comprehensive meta-analysis software (version 2) [24]. Funnel plots and Egger’s regression test will be used to examine the possible risk of publication bias. Heterogeneity among studies will be assessed by Cochran’s *Q χ*^2^ statistics and Higgins (*I*^2^ statistics) method [25]. *I*^2^ describes the percentage of total variations across the studies due to heterogeneity rather than chance. *I*^2^ value greater than 80% will be indicative of considerable heterogeneity [26]. In addition, the heterogeneity among studies will be checked manually in terms of study population, geographic distribution, and methods. The pooled prevalence and odds ratio (OR) of determinants of adherence will be generated using a weighted inverse-variance meta-analysis model. If substantial heterogeneity is detected, random-effects model results will be reported. Arcsine transformation will be carried out to minimize the effect of studies with very high or low effect size if normality assumption will be fulfilled [27]. The result will be presented using forest plots [28] and Harvest plots [29] when necessary. Qualitative analysis will be performed to construct a PRECEDE-PROCEED conceptual framework in the context of LMIC [10]. PRECEDE-PROCEED conceptual framework includes four groups of factors: predisposing factors (e.g., age, educational status, income, knowledge on anemia and prevention), enabling factors (e.g., number of iron/folate tablets received, acceptability of iron/folate supplements, sides effects), reinforcing factors (e.g., number of antenatal care visits, use of reminding techniques, support from family and relatives), and environmental factors (e.g., availability of antenatal care, access to antenatal care) [10].

### Sensitivity analysis

Leave-one-out Jackknife sensitivity analysis will be used to stabilize the variance of studies with very low or very large prevalence estimates [30, 31]. If the point estimate of the new pooled effect size is outside of the 95% confidence interval of the original/previous pooled effect size, it will be concluded that the excluded study has a significant effect on the pooled estimate. Thus, the study should not be included in the final analysis whether the effect of the study is too small or too large. In addition, the random- and fixed-effects model will be compared and decision will be made based on the best fitting model to the data.

### Subgroup analysis

Subgroup analysis will be carried out based on epidemiological and/or clinical covariates that can impact estimates such as study design, measures of adherence, age of women, residence of women, and geographical distribution.

### Potential methodological amendments

If protocol modifications are required, we will include the detailed description of any changes along with a justification during the publication of the review results.

## Discussion

The protocol has been registered and written in accordance with a standardized guideline which is helpful to replicate methods in other nations. Contemporary evidence on the prevalence and determinants of adherence in LMIC will be synthesized using an appropriate statistical method and qualitatively using PRECEDE-PROCEED conceptual framework [10]. Given that this is the first study, it would have substantial implications for researchers, clinicians, and policymakers in LMIC. We will develop a conceptual framework in the context of LMIC which could be helpful for prioritizing problems in primary healthcare and building statistical models for researchers in LMIC.

## Additional files

Additional file 1: PRISMA-P (Preferred Reporting Items for Systematic review and Meta-Analysis Protocols) 2015 checklist: recommended items to address in a systematic review protocol*. (DOCX 22 kb)

Additional file 2: PubMed search string. (DOCX 16 kb)

Additional file 3: JBI Data Extraction Form for Experimental/Observational Studies. (DOCX 80 kb)

## Abbreviations

JBI: Joanna Briggs Institute
LMIC: Low- and middle-income countries
NOS: Newcastle-Ottawa Scale
PECO: Population, exposure, comparator, and outcome
PROSPERO: Prospective Register of Systematic Review
RCTs: Randomized controlled trials
