# Supplementary material for: Adherence to prenatal iron-folic acid supplementation in low- and middle-income countries (LMIC): a protocol for systematic review and meta-analysis
Source: Syst Rev. 2018 Jul 25;7:107. doi: 10.1186/s13643-018-0774-x (PMC6060532; doi:10.1186/s13643-018-0774-x)
Supplement: Supplementary file 2 — PubMed search string. (DOCX 16 kb) [file 13643_2018_774_MOESM2_ESM.docx]

| **Component** | **Search terms** | **n hits****^¥^** |
| --- | --- | --- |
| #1 | ("Pregnancy"[Mesh] OR "Prenatal Care"[Mesh] OR "Pregnant Women"[Mesh] OR pregnan*[tiab] OR antenat*[tiab] OR prenat*[tiab]) | 968,897 |
| #2 | ("Folic Acid"[Mesh] OR iron[tiab] OR folic acid[tiab] OR folate[tiab] OR vitamin b9[tiab] OR "iron folate"[tiab] OR "iron folic acid"[tiab] OR "iron-folic acid"[tiab]) | 215,250 |
| #3 | ("Patient Compliance"[Mesh] OR complian*[tiab] OR adher*[tiab] OR utilization*[tiab] OR utilisation*[tiab] OR coverage[tiab] OR consumption[tiab] OR reported intake[tiab]) | 801,868 |
| #4 | #1 OR #2 OR #3 | 1,453 |

**PubMed search string**

***^¥^*** *=search date is June 25, 2018 at 5:11PM*
