# Supplementary material for: Adherence to prenatal iron-folic acid supplementation in low- and middle-income countries (LMIC): a protocol for systematic review and meta-analysis
Source: Syst Rev. 2018 Jul 25;7:107. doi: 10.1186/s13643-018-0774-x (PMC6060532; doi:10.1186/s13643-018-0774-x)
Supplement: Supplementary file 3 — JBI Data Extraction Form for Experimental/Observational Studies. (DOCX 80 kb) [file 13643_2018_774_MOESM3_ESM.docx]

**JBI Data Extraction Form for Experimental/Observational Studies**

Reviewer Author Journal

Date Year Record Number

**Study Method** RCT Quasi-RCT Longitudinal

**Participants**

Retrospective Observational Other

Setting Population Sample size

Intervention 1 Intervention 2 Intervention 3

**Interventions**

**Intervention 1:**

**Intervention 2:**

**Intervention 3:**

**Clinical outcome measures**

| Outcome Description | Scale/measure |
| --- | --- |
|  |  |
|  |  |
|  |  |
|  |  |
|  |  |

**Study results**

Dichotomous data

| Outcome | Intervention ( )  number / total number | Intervention ( )  number / total number |
| --- | --- | --- |
|  |  |  |
|  |  |  |
|  |  |  |
|  |  |  |
|  |  |  |

Continuous data

| Outcome | Intervention ( ) mean & SD (number) | Intervention ( ) mean & SD (number) |
| --- | --- | --- |
|  |  |  |
|  |  |  |
|  |  |  |

**Authors’ conclusions:**

**Comments:**
